# Soy Protein Concentrate Diets Inversely Affect LPS-Binding Protein Expression in Colon and Liver, Reduce Liver Inflammation, and Increase Fecal LPS Excretion in Obese Zucker Rats

**DOI:** 10.3390/nu16070982

**Published:** 2024-03-28

**Authors:** Wei Li, Reza Hakkak

**Affiliations:** 1 Department of Dietetics and Nutrition, University of Arkansas for Medical Sciences, Little Rock, AR 72205, USA; wli@uams.edu; 2 Arkansas Children’s Research Institute, Little Rock, AR 72202, USA; 3 Department of Pediatrics, University of Arkansas for Medical Sciences, Little Rock, AR 72205, USA

**Keywords:** soy protein concentrate, isoflavone, liver inflammation, lipopolysaccharide, lipopolysaccharide-binding protein, obese Zucker rats

## Abstract

Dietary soy protein and soy isoflavones have anti-inflammatory properties. Previously, we reported that feeding soy protein concentrate diet (SPC) with low or high isoflavone (LIF or HIF) to young (seven-week-old) obese (fa/fa) Zucker rats inhibits lipopolysaccharide (LPS) translocation and decreases liver inflammation compared to a casein control (CAS) diet. The current study investigated whether SPC-LIF and SPC-HIF diets would reduce liver inflammation in adult obese Zucker rats fed a CAS diet. A total of 21 six-week-old male obese (fa/fa) Zucker rats were given CAS diet for 8 weeks to develop obesity then randomly assigned to CAS, SPC-LIF, or SPC-HIF (seven rats/group) diet for an additional 10 weeks. The expression of LPS-translocation, inflammation, and intestinal permeability markers were quantified by qPCR in liver, visceral adipose tissue (VAT), and colon. LPS concentration was determined in both the colon content and fecal samples by a Limulus amebocyte lysate (LAL) test. SPC-LIF and SPC-HIF diets significantly decreased liver LPS-binding protein (LBP) expression compared to CAS diet (*p* < 0.01 and *p* < 0.05, respectively). SPC-HIF diet also significantly decreased liver MCP-1 and TNF-α expression (*p* < 0.05) and had a trend to decrease liver iNOS expression (*p* = 0.06). In the colon, SPC-HIF diet significantly increased LBP expression compared to CAS diet (*p* < 0.05). When samples from all three groups were combined, there was a negative correlation between colon LBP expression and liver LBP expression (*p* = 0.046). SPC diets did not alter the expression of intestinal permeability markers (i.e., occludin, claudin 3, and zonula occludens-1) in the colon or inflammation markers (i.e., TNF-α and iNOS) in VAT or the colon. LPS levels in the colon content did not differ between any groups. Fecal LPS levels were significantly higher in the SPC-LIF and SPC-HIF groups compared to the CAS group (*p* < 0.01). In conclusion, SPC, particularly SPC with HIF, reduces liver LBP expression and inflammation makers (i.e., TNF-α and MCP-1 expression) in adult obese Zucker rats, likely by reducing LPS translocation.

## 1. Introduction

With over half of the global population projected to be obese by 2035, obesity remains a pressing public health concern [1]. Emerging data from 2019–2023 also suggest that the risk of obesity was exacerbated by the coronavirus infectious disease 2019 (COVID-19) pandemic and its associated lockdown measures in adults, adolescents, and children worldwide [2]. Obesity increases the risk of developing comorbidities such as type II diabetes, cardiovascular diseases, metabolic dysfunction-associated steatotic liver disease (MASLD) (previously known as nonalcoholic fatty liver disease (NAFLD)), and certain types of cancer that reduce quality of life and increase the burden of the healthcare system [1,3]. Although the etiologies of these chronic diseases are multifactorial, a common paradigm is a low-grade systemic inflammation. During obesity, adipose tissue is increasingly infiltrated by bone marrow-derived immune cells, particularly macrophages, that, together with dysfunctional adipocyte, secrete proinflammatory cytokines and adipokines into the systemic circulation, leading to a wide range of metabolic dysregulations in other organs/tissues [4].

In addition to the dysfunctional adipose tissue, systemic inflammation may also arise from the gastrointestinal (GI) tract. The GI tract maintains a delicate balance that allows the absorption of nutrients and dietary compounds while safeguarding the host from over-exposure to the commensal microbes that may trigger undesired inflammatory response. The impact of the gut microbiota on health and diseases has been extensively studied and well appreciated in recent years [5,6,7]. The commensal microbes interact with both the diet and the host, help shape the body function and immune response, and exert impact beyond the GI tract. A well-demonstrated mechanism by which a dysfunctional GI tract leads to systemic inflammation is the passage of the bacterial component lipopolysaccharide (LPS) across the intestinal barrier, traversing the portal vein into the liver, a process known as LPS translocation [8]. Although the liver has the capacity to remove LPS, excessive LPS translocation leads to liver inflammation and fatty liver diseases [9]. Furthermore, excessive LPS translocation may elevate LPS levels in the systemic blood, known as metabolic endotoxemia (ME). ME is a main cause of low-grade systemic inflammation and is associated with obesity and its comorbidities [10,11,12].

Because dietary compounds directly interact with both host GI tract cells and microbiota, dietary interventions have been explored as a strategy to reduce LPS translocation and ME by promoting GI tract barrier function and/or optimizing microbiota composition [13]. Consuming soy foods may reduce chronic disease risks, and soy’s health benefits have been widely attributed to its high-quality protein and isoflavone compounds [14,15,16]. The benefit of soy foods and soy isoflavones to GI tract health and microbiota composition is supported by epidemiological evidence and studies in both humans and animals; however, further research is needed to investigate the effects of specific types of soy foods and soy ingredients, such as textured soy proteins [17]. Soy protein concentrate (SPC) is produced by defatting soy meal through water extraction and can be used as an ingredient to enrich other food products [18]. Isoflavones are natural compounds in soy. The concentration of isoflavone in soy protein products is affected by the processing methods and may range from around 2 mg/g protein to less than 1 mg/g protein [19]. Soy isoflavones are mainly in glycoside forms as daidzin and genistin, with a small amount in aglycone forms as daidzein and genistein [20,21].

We are particularly interested in understanding the mechanism of the benefit of soy protein and soy isoflavones in reducing liver inflammation. Previously, we reported that feeding young (seven-week-old, prior to the onset of obesity) fa/fa Zucker rats soy protein concentrate (SPC) with low or high isoflavone (LIF or HIF) for 18 weeks during the development of obesity reduced liver LPS staining and liver expression of LBP and proinflammatory proteins including TNF-α, MCP-1, and iNOS without reducing body mass [22]. However, it is not clear whether SPC-LIF and SPC-HIF diets would reduce liver inflammation in adult Zucker rats that have already developed obesity. We hypothesized that SPC diets would provide similar anti-inflammatory benefits in adult obese Zucker rats. This study was designed to test this hypothesis and further explore the effect of SPC-LIF and SPC-HIF diets on LPS levels in colon content and fecal samples, inflammation markers in the visceral adipose tissue (VAT), as well as inflammation and epithelial permeability indicators in the colon.

## 2. Materials and Methods

### 2.1. Ethics Statement

The animal care protocol and procedures in the study were approved by the University of Arkansas for Medical Sciences and Arkansas Children’s Research Institute Institutional Animal Care and Use Committee (Protocol code no. 3968; approved on 20 December 2019) and followed the guidelines of the United States Department of Agriculture (USDA, Washington, DC, USA) Animal Welfare Act.

### 2.2. Experimental Design

A total of 21 six-week-old male obese (fa/fa) Zucker rats were purchased from Charles River Laboratories (Wilmington, MA, USA) and were fed a casein protein control (CAS) diet for 8 weeks to develop obesity before being randomly assigned to three dietary groups (7 rats per group) to receive a soy protein concentrate (SPC) diet with low isoflavone (LIF), an SPC diet with high isoflavone (HIF), or the CAS diet for additional 10 weeks. The SPC-LIF diet was included to test whether any observed effect was due to the protein or the isoflavone content of SPC. All diets were prepared by Archer Daniels Midland (AMD) (Decatur, IL, USA) with detailed compositions reported previously [23]. The SPC-LIF diet had 0.154 mg isoflavone/g protein with an aglycone component of approximately 0.16 mg/g protein. The SPC-HIF diet had 2.153 mg isoflavone/g protein with an aglycone component of approximately 1.72 mg/g protein. Isoflavone levels were below detection in the CAS diet. To balance amino acid profile, L-cystine was added at 3 g/kg to the casein diet and 1.2 g/kg to SPC-LIF and SPC-HIF diets and L-methionine was added at 2.2 g/kg to SPC-LIF and SPC-HIF diets. Diets were isocaloric and isonitrogenous [23]. After 10 weeks of feeding one of the three diets (CAS, SPC-LIF, or SPC-HIF), rats were anesthetized with carbon dioxide and euthanized by decapitation. Liver, colon, visceral adipose tissue (VAT), and fecal samples were collected and snap frozen in liquid nitrogen and stored at −80 °C for subsequent analyses, as described previously [22,24].

### 2.3. Quantitation of mRNA Expression by Quantitative PCR (qPCR)

Total RNA was extracted from frozen liver, colon, and visceral adipose tissue (VAT) samples using TRIzol Reagent (ThermoFisher Scientific, 15596026, Waltham, MA, USA). For liver and colon samples, the protocol provided by the manufacturer was followed. For VAT samples, a modified protocol for isolating RNA from adipose tissue was used [25]. The modified protocol included an additional centrifugation step to remove the top fatty layer from TRIzol homogenates, higher centrifugation speed, and increased number of washes of precipitated RNA pellets with 75% ethanol to better remove lipid contaminations. Isolated RNA samples were treated with DNase I (ThermoFisher Scientific, EN0521) to eliminate potential genomic DNA contamination. A SuperScript™ First-Strand Synthesis System for RT-PCR (ThermoFisher Scientific, 11904018) was used to generate cDNA. qPCR was performed using validated TaqMan assays (ThermoFisher Scientific) (Table 1), and relative expression levels were normalized to the expression of 18s rRNA by delta-delta Ct (ΔΔCt) method. A no reverse transcriptase negative control from the RT-PCR step was included in each qPCR experiment. All qPCR experiments were performed on a QuantStudio 6 real-time PCR system (Applied Biosystems, Foster City, CA, USA).

### 2.4. Lipopolysaccharide (LPS) Quantification Using Limulus Amebocyte Lysate (LAL) Test

Colon content was isolated by cutting frozen colon samples longitudinally and peeling open the colon wall. Fecal samples were collected over a 12 h period at the end of the experiment. The weights of colon content samples and fecal samples were recorded before homogenization in 1 mL endotoxin-free Dulbecco’s PBS (Sigma-Aldrich, TMS-012-A, St Louis, MI, USA). To quantify LPS in colon content and fecal samples, LAL assays were performed using a chromogenic endotoxin quant kit (ThermoFisher Scientific, A39552). The concentrations of LPS in the homogenates were used to calculate the amount of LPS (endotoxin units (EU)) per gram of sample.

### 2.5. Statistical Analysis

Body mass data were analyzed by mixed ANOVA. qPCR data and LAL assay data were log-transformed and analyzed using one-way ANOVA followed by Fisher’s LSD post hoc tests. The correlation between colon LBP expression and liver LBP expression was analyzed by a simple linear regression. Statistical significance was determined at the *p* < 0.05 level in all tests. Analyses were performed in SPSS Statistics for Windows, version 28.0.0.0 (IBM Corp., Armonk, NY, USA).

## 3. Results

### 3.1. Body Mass of CAS, SPC-LIF, and SPC-HIF Diet-Fed Rats

**Figure 1:**
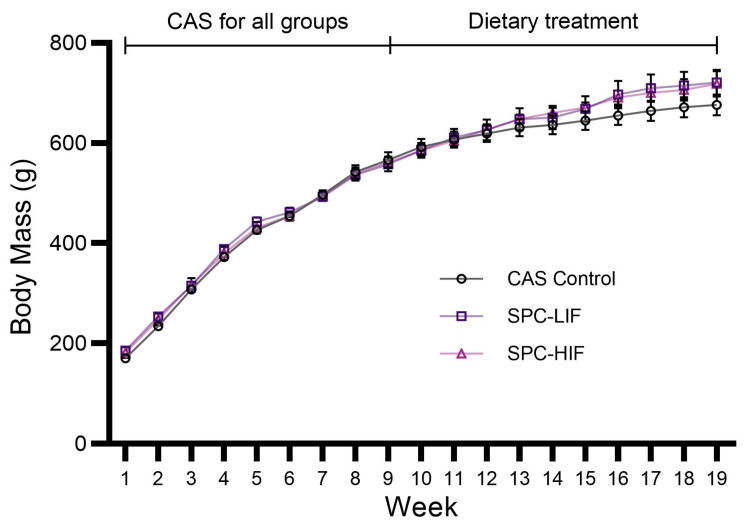
Body mass (BM) of rats over the course of the experiment. Rats were fed a casein control diet (CAS control) for 8 weeks to develop obesity and then randomly assigned to be fed a CAS diet, a soy protein concentrate diet with low isoflavone (SPC-LIF), or a soy protein concentrate diet with high isoflavone (SPC-HIF) for 10 additional weeks. Data are expressed as mean ± S.E.

Rat body mass (BM) data from the beginning of the experiment (Week 1) to the end (Week 19) of dietary treatment (CAS control diet, SPC-LIF diet, or SPC-HIF diet) are shown in Figure 1. All groups of rats gained BM over the course of the experiment. BM did not significantly differ between any dietary groups.

### 3.2. The Effect of SPC-LIF and SPC-HIF Diets on Liver Gene Expression

**Figure 2:**
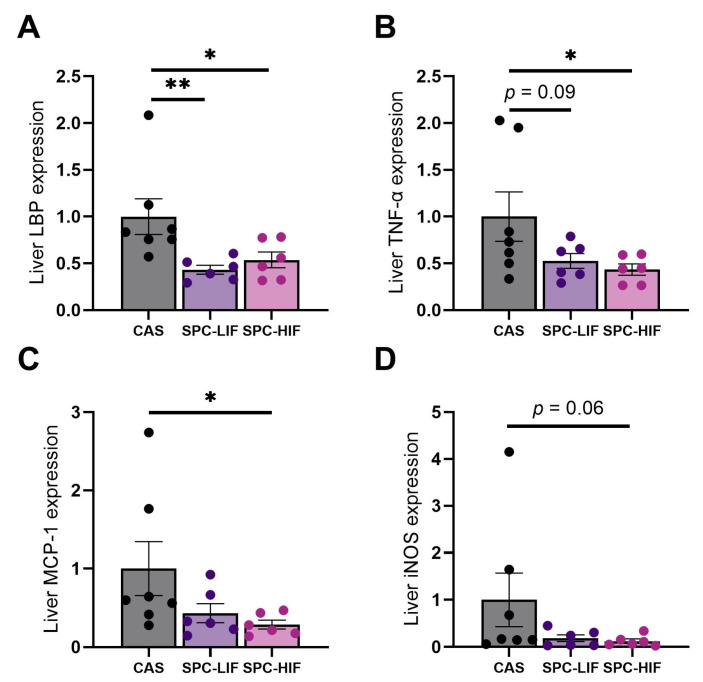
Liver expression levels (mean ± S.E.) of lipopolysaccharide-binding protein (LBP) (**A**), tumor necrosis factor α (TNF-α) (**B**), monocyte chemoattractant protein 1 (MCP-1) (**C**), and inducible nitric oxide synthase (iNOS) (**D**) in obese Zucker rats fed CAS, SPC-LIF, and SPC-HIF diets. Data are expressed as ratios to the CAS control group. * *p* < 0.05, ** *p* < 0.01 in post hoc tests between two groups.

After the obese Zucker rats were fed CAS, SPC-LIF, or SPC-HIF diet for 10 weeks, LBP expression was significantly lower in both SPC-LIF and SPC-HIF groups compared to the CAS control (Figure 2A). The expression levels of TNF-α and MCP-1 were significantly lower in SPC-HIF diet-fed rats compared to CAS-fed rats (Figure 2B,C). There was also a trend of less iNOS expression in SPC-HIF-fed rats (Figure 2D) and less TNF-α expression in SPC-LIF diet-fed rats compared to CAS diet-fed rats (Figure 2B). Because LBP is a surrogate marker for LPS translocation [26], and increased liver LBP expression was accompanied by increased liver LPS staining in our previous study [22], these results suggest that both SPC-LIF and SPC-HIF diet reduced LPS translocation, resulting in decreased liver inflammation, particularly in the SPC-HIF group.

### 3.3. The Effect of SPC-LIF and SPC-HIF Diets on Colon Gene Expression

**Figure 3:**
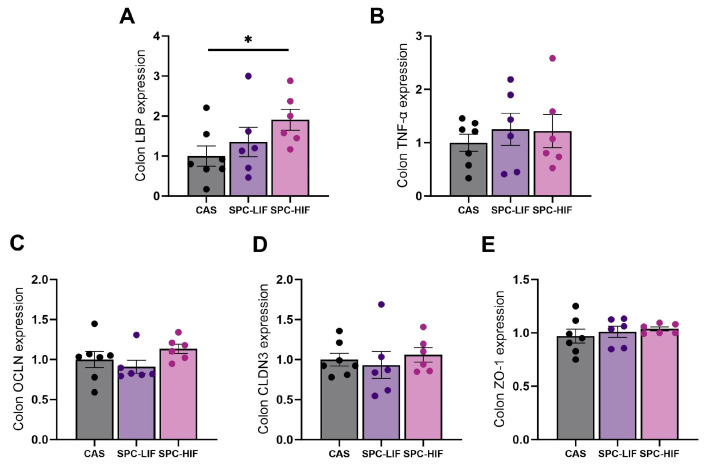
Colon expression levels (mean ± S.E.) of LBP (**A**), TNF-α (**B**), occludin (OCLN) (**C**), claudin 3 (CLDN3) (**D**), and Zonula Occludens-1 (ZO-1) (**E**) in obese Zucker rats fed CAS, SPC-LIF, and SPC-HIF diets. Data are expressed as ratios to the CAS control group. * *p* < 0.05 in post hoc tests between two groups.

Because colon bacteria are the main source of LPS, to determine whether the differences in liver LBP and inflammatory gene expression between SPC diets and CAS control was a result of differences in LPS originating from the colon or differences in colon permeability, we measured the expression of LBP, TNF-α, and colon permeability markers occludin (OCLN), claudin 3 (CLDN3), and Zonula Occludens-1 (ZO-1). Counterintuitively, SPC-HIF diet-fed rats had significantly higher levels of LBP expression in the colon compared to CAS control diet-fed rats (Figure 3A). There were no differences in expression levels of TNF-α or epithelium permeability markers in the colon between any groups (Figure 3B–E).

### 3.4. The Correlation between Colon and Liver LBP Expression

**Figure 4:**
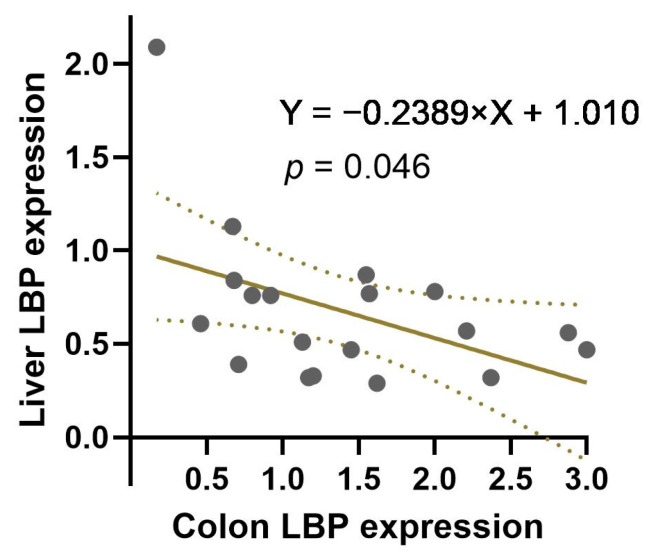
The negative correlation between liver LBP expression and colon LBP expression in obese Zucker rats (*p* = 0.046) when LBP expression data from CAS, SPC-LIF, and SPC-HIF groups are combined. Each circle represents an individual rat. Dashed lines indicate 95% confidence interval.

Since SPC diets appeared to have opposite effects on liver LBP expression and colon LBP expression, we analyzed liver and colon LBP expression by linear regression after combining data from all three dietary groups. There was a significantly negative correlation between liver LBP expression and colon LBP expression (Figure 4).

### 3.5. The Effect of SPC-LIF and SPC-HIF Diets on LPS Concentration in Colon Content and Fecal Samples

**Figure 5:**
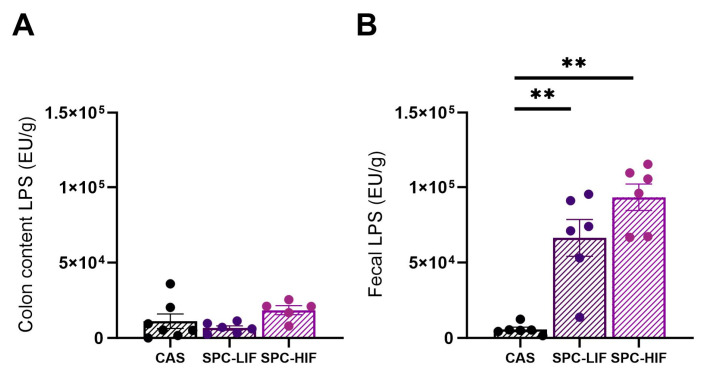
LPS concentration (mean ± S.E., EU/g) determined in colon content (**A**) and feces (**B**) by Limulus amebocyte lysate (LAL) endotoxin assay in CAS, SPC-LIF, and SPC-HIF diet-fed obese Zucker rats. ** *p* < 0.01 in post hoc tests between two groups.

Next, we measured LPS concentrations in content dissected from the colon lumen and in fecal samples. LPS concentrations of colon content did not differ between any groups (Figure 5A). In contrast, fecal samples of SPC-LIF and SPC-HIF diet-fed rats had significantly higher LPS concentrations compared to CAS diet-fed rats (Figure 5B).

### 3.6. The Effect of SPC-LIF and SPC-HIF Diets on VAT Gene Expression

**Figure 6:**
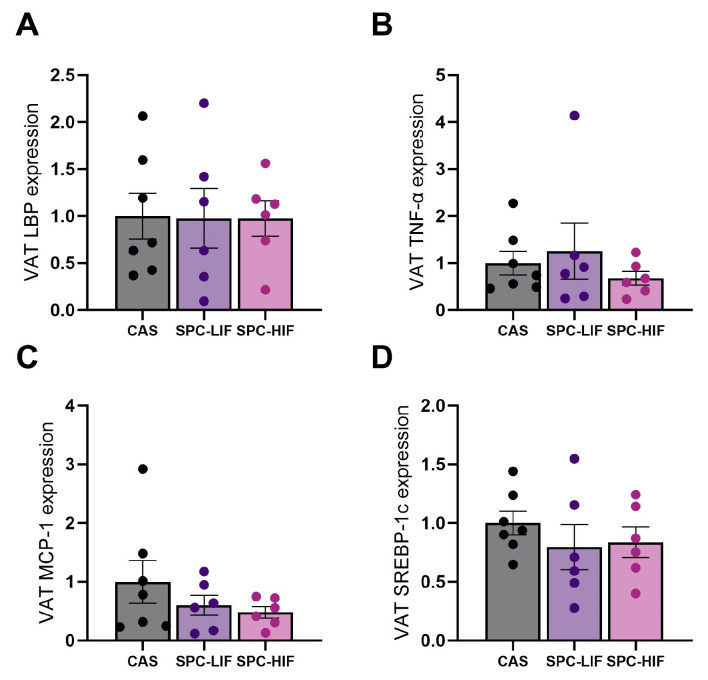
Visceral adipose tissue (VAT) expression levels (mean ± S.E.) of LBP (**A**), TNF-α (**B**), MCP-1 (**C**), and SREPB-1c (**D**) in obese Zucker rats fed CAS, SPC-LIF, and SPC-HIF diets. Data are expressed as ratios to the CAS control group.

Because of the close proximity of VAT to the liver and GI tract, we measured LBP, TNF-α, and MCP-1 expression in VAT samples to determine any potential involvement of VAT in LPS-mediated inflammation and the anti-inflammatory effect of SPC. There were no differences in LBP, TNF-α, or MCP-1 expression in VAT between any groups (Figure 6A–C). We also measured the expression of a lipogenesis marker SREBP-1c in VAT and detected no significant differences in SREBP-1c expression levels between any groups (Figure 6D).

## 4. Discussion

Obesity is associated with increased LPS translocation from the GI tract to the liver and the systemic circulation, and the subsequent low-grade systemic inflammation mediates many comorbidities of obesity [11,27,28]. Previous, we reported that feeding young (7-week-old) Zucker fa/fa rats SPC-LIF diet or SPC-HIF diet for 18 weeks reduced LBP expression and LPS staining in the liver and decreased liver inflammation compared to a CAS control diet [22]. The current study was designed to investigate whether soy protein concentrate would provide a similar benefit of reducing liver inflammation in adult obese Zucker rats with existing obesity. We found that feeding SPC diets to adult obese Zucker rats decreased liver LBP expression, and the SPC-HIF diet also attenuated liver TNF-α and MCP-1 expression compared to the CAS control diet. The lack of significant difference in TNF-α and MCP-1 expression between SPC-LIF diet and CAS diet groups suggests that although the effect on liver gene expression may have been provided mainly by soy protein, soy isoflavones may offer additional benefits.

The GI tract safeguards the body from inflammatory stimuli from bacteria-derived compounds by mechanisms that include the tight junctions between epithelial cells, the intestinal alkaline phosphatase (IAP) produced by epithelial cells, the inner and outer mucus layers lining the lumen, and the anti-microbial proteins secreted from Paneth cells [8]. In both human studies and animal models, obesity is associated with compromised GI tract defense mechanisms as well as dysbiosis of the gut microbiota that together allow increased LPS passage into the liver and systemic circulation [29,30]. Macrophages are the most abundant immune cells in the liver and play a crucial role in regulating liver inflammation and immune response [31]. LPS translocation exacerbates liver inflammation by triggering macrophages to release inflammatory cytokines including TNF-α [32,33]. The contribution of hepatic inflammation to chronic diseases such as MASLD (previously known as NAFLD) is supported by the experimental evidence that TNF-α-/- mice and mice treated with a TNF-α receptor antagonist are resistant to hepatic steatosis [34,35].

Because the reduced liver LBP, TNF-α, and MCP-1 expression in SPC-HIF diet-fed obese rats may be a result of the SPC-HIF diet promoting better intestinal barrier function and/or optimizing the gut microbiota to produce less LPS, we went on to quantify the expression of tight junction proteins occludin, claudin 3, and ZO-1 in colon tissue and detected no difference between SPC diets and CAS control diet-fed obese rats (Figure 3C–E). Therefore, we reasoned that the differences in liver inflammation may not be due to differences in intestinal permeability but rather a result of less LPS originating from the gut bacteria in SPC diet-fed obese rats. To test this hypothesis, we measured LPS concentration in colon lumen content and fecal samples. There were no differences in LPS concentration in the colon lumen content between any dietary groups (Figure 5A). Surprisingly, LPS concentrations in fecal samples from SPC-LIF and SPC-HIF diet-fed obese rats were 8–10-fold higher compared to those in fecal samples from CAS diet-fed obese rats (Figure 5B). Consuming soy foods can alter gut microbiota composition in humans and animal models [36]. Existing studies using surrogate markers such as serum LBP level or gut Firmicutes to Bacteroidetes ratios did not have consistent results, reporting LPS production by gut bacteria may either increase or decrease in response to soy [37,38]. No existing studies have directly measured fecal or intestinal content LPS level in response to soy foods in humans or animals. To our knowledge, this study not only was the first to directly measure LPS level in the colon content and fecal samples in response to soy protein but also suggested that SPC diet-induced LPS changes were dependent on the sampling site (i.e., colon content vs. feces).

The seemingly contradicting LPS level comparison results between dietary groups observed in colon content and fecal samples in the current study may point to a novel protective effect of soy protein, in which consuming soy protein decreases LPS/LPS-producing bacteria adhesion and facilitates their fecal excretion. The GI tract is protected by mucus secreted from goblet cells. The mucus layer(s) is especially thick in the colon where a large number of microbes habituate [39]. In our experiment, when colon content was sampled for LPS measurement, the samples likely contained the mucus layers along with the bacteria and other content that is not associated with the mucus. In contrast, the excreted fecal samples likely had less mucus content. Perhaps the bacteria distribution pattern between the colon mucus layer and the colon lumen were differently affected by SPC diets and CAS diet, resulting in less adhesion of LPS as well as LPS-producing bacteria to the colon wall of SPC diet-fed obese rats and higher LPS excretion in the feces.

In the current study, we observed a significantly higher level of colon LBP expression in the SPC-HIF group compared to the control group (Figure 3A) and a negative correlation between colon LBP expression and liver LBP expression when all groups of animals were combined (Figure 4). At first, we thought the higher level of colon LBP expression may be a result of a higher concentration of LPS in the colon content of the SPC-HIF groups. However, the colon content LPS concentration was not different between any groups (Figure 5A), neither was the expression of the inflammation marker TNF-α in the colon (Figure 3B). These results suggest that the higher level of colon LBP expression in SPC-HIF-fed rats was not a result of increased LPS exposure, nor was it associated with elevated colon inflammation; rather, given the negative correlation between colon and liver LBP expression, a higher colon LBP expression appears to be a protective mechanism that reduces LPS translocation. Although the liver is the principal organ for LBP expression and secretion, LBP is secreted by enterocytes. A previous study reported that the human colon cell line Caco-2 cells secrete LBP, and LBP is also detectable in the intestinal mucus of mice [40]. LBP is a double-edge sword during LPS exposure by facilitating LPS-triggered inflammatory response at basal concentrations while neutralizing LPS at high concentrations [41]. SPC-HIF-fed obese rats likely secreted more LBP into the mucus layers and the lumen of the colon, preventing LPS and LPS-producing bacteria from entering into proximity of the epithelial cells. This potential protective effect of SBP in the colon may also explain the significantly higher fecal LPS levels in the SPC diet groups, as LPS fecal excretion may be facilitated by LBP.

A limitation of the current study is the use of colon tight junction proteins occludin, claudin 3, and ZO-1 expression levels as a surrogate marker for colon epithelium permeability. Although we did not detect significant differences in colon tight junction protein expression between dietary groups, we cannot rule out potential functional differences in GI barriers. Future functional assays are needed to test this possibility.

In humans, soy consumption may lead to some side effects, with the most common including constipation and diarrhea [42]. We did not observe similar side effects in the obese Zucker rats from the current study. Moreover, soy may also pose health risk to individuals with iodine deficiency or certain types of cancers [42]. Although those conditions are not directly relevant to this study, considerations need to be taken in future studies that investigate potential similar beneficial effects of SPC in humans with obesity.

## 5. Conclusions

A soy protein concentrate diet with low or high isoflavone can reduce obesity-associated liver inflammation in adult obese Zucker rats, likely by inhibiting LPS translocation. Future studies may provide new information on whether a similar protective mechanism by soy protein exists in humans with adulthood obesity.

## Figures and Tables

**Table 1:** Quantitative PCR assay list.

Gene Name	Species	Assay ID (ThermoFisher)
Tnfa	Rat	Rn01525859_g1
Ccl2/Mcp1	Rat	Rn00580555_m1
iNos/Nos2	Rat	Rn00561646_m1
Lbp	Rat	Rn00567985_m1
Srebp1c	Rat	Rn01495769_m1
Ocln	Rat	Rn00580064_m1
Cldn3	Rat	Rn00581751_s1
Zo-1/Tjp1	Rat	Rn02116071_s1
18s rRna	Rat	Rn03928990_g1

## Data Availability

Data can be shared upon reasonable request to the corresponding author.
